# Efficacy of tocilizumab treatment in severely ill COVID-19 patients

**DOI:** 10.1186/s13054-020-03224-7

**Published:** 2020-08-27

**Authors:** Jie Zhao, Wei Cui, Bao-ping Tian

**Affiliations:** Department of Critical Care Medicine, The Second Affiliated Hospital, Zhejiang University School of Medicine, 88 Jiefang Rd., Hangzhou, 310009 Zhejiang China

**Keywords:** Tocilizumab, COVID-19, Efficacy, Meta-analysis

The current coronavirus disease 2019 (COVID-19) pandemic induced by severe acute respiratory syndrome coronavirus 2 (SARS-CoV-2) has already caused a global increase in hospitalizations and deaths. Unfortunately, effective medicines to fight this disease, especially in the severely ill patients, are still lacking [1]. Tocilizumab, a humanized monoclonal antibody used in rheumatoid arthritis treatment, might also be effective in treating severe COVID-19 as it could selectively target the interleukin-6 (IL-6) receptor [2]. Considering the uncertain efficacy of tocilizumab treatment in severe COVID-19, we conducted a systematic review and meta-analysis to clarify this added effect of tocilizumab.

We performed a systematic search of PubMed, Embase, Medline, Cochrane, and CNKI database through 25 July 2020, using the following search terms alone or in combination: (1) “COVID-19,” (2) “coronavirus,” (3) “SARS-CoV-2,” (4) “COVID,” (5) “anti-interleukin-6 receptor antibodies,” (6) “anti-IL-6 receptor antibodies,” (7) “anti-IL-6,” (8) “tocilizumab,” (9)“sarilumab,” and (10) “siltuximab.” Clinical trials regarding tocilizumab as a therapeutic intervention were selected. Two independent investigators selected eligible trials and extracted data from articles. Discrepancies in screening/data extraction were addressed by group discussion. Proportional variables were measured by odds ratio (OR) and corresponding 95% confidence intervals (CI). *P* values < 0.05 were considered statistically significant. Significant heterogeneity (*P* < 0.10 or *I*^2^ ≥ 50%) was evaluated by chi-square and *I*^2^ tests in a fixed-effect model. The comparison of the outcome between tocilizumab and control was conducted by using Review Manager 5.4 (Revman, The Cochrane Collaboration, Oxford, UK).

Finally, 10 studies involving 1675 severe COVID-19 patients were included, among which only one trial was a randomized controlled trial, while the rest were all retrospective cohort studies. These studies included COVID-19 patients who were older/elderly (mean/median age ≥ 52 years) in America, Europe, and India, among whom 675 patients received tocilizumab, while 1000 patients underwent standard care. Severe COVID-19 patients received tocilizumab via intravenous or subcutaneous formulation, while doses and administration time points varied. Standard care included hydroxychloroquine, lopinavir/ritonavir, remdesivir, azithromycin, low weight heparin, and/or methylprednisolone, among others **(**Table 1**)**. Our meta-analysis result revealed a significant difference in mortality between tocilizumab group (132/675, 19.5%) and control group (283/1000, 28.3%) in the fixed-effect model (OR, 0.47; 95%Cl, 0.36–0.60; *P* < 0.00001), suggesting efficacy of tocilizumab treatment for severe COVID-19. However, high heterogeneity was also observed (*I*^2^ = 74%, *P* < 0.0001) as shown in Fig. 1. SARS-CoV-2 infection might cause a hyperimmune response associated with acute respiratory distress (ARDS), the latteris a leading cause of death for severe COVID-19 [3]. Uncontrolled immune activation would result in cytokine storm, also known as cytokine release syndrome (CRS), appearing as overproduction of pro-inflammatory cytokines and chemokines [4]. Severe COVID-19 patients always present elevated inflammatory markers, among which the elevation of IL-6 is associated with severity of COVID-19 [5]. Besides, the upregulated expression of IL-6 receptor (IL-6R) was also detected in COVID-19 patients [6]. Therefore, IL-6/IL6R might serve as a messenger not only for transmitting inflammatory signals throughout the lung and other organs but also by activating cellular signal pathway, thus causing ARDS and multiple organ dysfunction. It is reasonable to speculate that IL-6 blockade is beneficial for avoiding poor prognosis.

**Table 1:** Study characteristics and demographics of included severely ill coronavirus disease 2019 (COVID-19) patients

Article	Study design	Country	Total patients	Mean/median age (years)	Standard care	Tocilizumab treatment	Patients category	Primary outcomes
Campochiaro C Eur J Intern Med 2020	Single-center retrospective cohort study	Italy	65	60 (control) 64 (tocilizumab)	Hydroxychloroquine, lopinavir/ritonavir, ceftriaxone, azithromycin	First intravenous 400 mg, second 400 mg was administered due to progressive respiratory worsening	Severe COVID-19 patients with hyper-inflammatory features admitted outside ICU requiring NIV and/or high-flow supplemental O_2_	Safety, efficacy
Capra R Eur J Intern Med 2020	Retrospective observational study	Italy	85	70 (control) 63 (tocilizumab)	Hydroxychloroquine, lopinavir/ritonavir	Tocilizumab once within 4 days	COVID-19-related pneumonia and respiratory failure, not needing mechanical ventilation	Survival rate
Colaneri M Microorganisms 2020	Retrospective case-control study	Italy	112	64 (control) 62 (tocilizumab)	Hydroxychloroquine, azithromycin, low weight heparin, methylprednisolone	First administration was 8 mg/kg (up to a maximum 800 mg per dose) intravenously, repeated after 12 h	Critically ill patients with severe COVID-19 pneumonia	Admission to the ICU and 7-day mortality rate
Gokhale Y EClinicalMedicine 2020	Retrospective cohort study	India	161	55 (control) 52 (tocilizumab)	Antibiotics, hydroxychloroquine oseltamivir, low molecular weight heparin, methylprednisolone	A single intravenous dose of 400 mg	COVID-19 with oxygen saturation of 94% or less despite giving supplemental oxygen of 15 L/min via non-rebreathing mask or PaO2/FiO2 ratio of less than 200	Death
Guaraldi G Lancet Rheumatol 2020	Retrospective observational cohort study	Italy	544	69 (control) 64 (tocilizumab)	Oxygen supply to target SaO_2_ reaching at least 90%, hydroxychloroquine, azithromycin at the physician’s discretion when suspecting a bacterial respiratory super-infection, lopinavir–ritonavir or darunavir–cobicistat, low molecular weight heparin	Intravenous tocilizumab was administered at 8 mg/kg bodyweight (up to a maximum of 800 mg) administered twice, 12 h apart; the subcutaneous formulation was used when there was a shortage of the intravenous formulation, at a dose of 162 mg administered in two simultaneous doses, one in each thigh	Severe pneumonia defined at least one of the following: presence of a respiratory rate of 30 or more breaths per minute, peripheral blood SaO_2_ of less than 93% in room air, a ratio of PaO_2_ to FiO_2_ of less than 300 mmHg in room air, and lung infiltrates of more than 50% within 24–48 h, according to Chinese management guidelines for COVID-19	Death or invasive mechanical ventilation
Klopfenstein T Med Mal Infect 2020	Retrospective case-control study	France	45	71 (control) 77 (tocilizumab)	Hydroxychloroquine or lopinavir-ritonavir, antibiotics, less commonly corticosteroids	1 or 2 doses (no detail was reported)	All critically COVID-19 patients in tocilizumab group, fewer critically ill patients in control	Death and/or ICU admissions
Moreno-Pérez O J Autoimmun 2020	Retrospective cohort study	Spain	236	57 (control) 62 (tocilizumab)	No detail was reported	Initial 600 mg, with a second or third dose (400 mg) in case of persistent or progressive disease	Severe COVID-19 pneumonia	All-cause mortality
Potere N Ann Rheum Dis 2020	Retrospective case–control study	Italy	80	54 (control) 56 (tocilizumab)	Hydroxychloroquine, darunavir/cobicistat, lopinavir/ritonavir, systemic corticosteroid	324 mg given as two concomitant subcutaneous injections	Severe COVID-19 pneumonia with hypoxemia (oxygen saturation < 90% on room air) requiring supplemental oxygen through nasal cannulas or mask	Requirement of IMV or death
Rojas-Marte GR QJM: An International Journal of Medicine 2020	Retrospective, case–control, single-center study	USA	193	62 (control) 59 (tocilizumab)	Hydroxychloroquine, azithromycin, corticosteroids anticoagulation, remdesivir, antibiotics for suspected bacterial infections, vasopressors	No detail was reported	Adult patients hospitalized with severe COVID-19	Overall mortality rate
Somers EC Clin Infect Dis 2020	Randomized controlled trial	USA	154	60 (control) 55 (tocilizumab)	Hydroxychloroquine, remdesivir, NSAIDs, ACEI/ARB, vasopressors, anticoagulation corticosteroid	The standard tocilizumab dose was 8 mg/kg (maximum 800 mg) × 1, additional doses were discouraged	Severe COVID-19 patients requiring mechanical ventilation	Survival probability after intubation

**Fig. 1:**
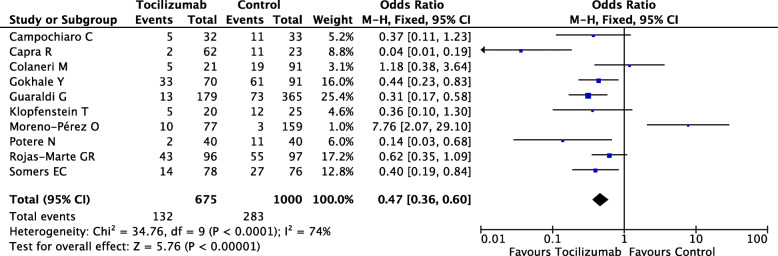
Forest plot of pooled mortality of severely ill coronavirus disease 2019 (COVID-19) patients from included studies

Our meta-analysis had several limitations: (1) most included studies were retrospective analysis of cases, resulting in poor quality of the included studies; (2) the uniformity of the diagnostic criteria for severe COVID-19 needs to be improved, and the extraction of related factors is limited; and (3) extraction of the original data is incomplete, and some data cannot be converted due to the lack of relevant data.

In summary, this is the first meta-analysis demonstrating the efficacy of tocilizumab treatment in severely ill COVID-19 patients.

## Data Availability

All data generated or analyzed during this study are included in this published article.

## Abbreviations

ACEI: Angiotension converting enzyme inhibitors
ARB: Angiotension receptors blockers
ARDS: Acute respiratory distress syndrome
COVID-19: Coronavirus disease 2019
CI: Confidence intervals
CRS: Cytokine release syndrome
FiO_2_: Fraction of inspired oxygen
ICU: Intensive care unit
IL-6: Interleukin-6
IMV: Invasive mechanical ventilation
NSAID: Non-steroidal anti-inflammatory drugs
NIV: Non- invasive Ventilation
OR: Odds ratio
PaO_2_: Partial pressure of oxygen
SaO_2_: Oxygen saturation
SARS-CoV-2: Severe acute respiratory syndrome coronavirus 2
